# Employment status and perceived health in the Hordaland Health Study (HUSK)

**DOI:** 10.1186/1471-2458-6-219

**Published:** 2006-08-29

**Authors:** Simon Overland, Nicholas Glozier, John Gunnar Mæland, Leif Edvard Aarø, Arnstein Mykletun

**Affiliations:** 1 Research Centre for Health Promotion, University of Bergen, Norway; 2 Division of Psychological Medicine, Institute of Psychiatry, Kings College London, UK; 3 The Department of Public Health and Primary Health Care, University of Bergen, Norway; 4 Norwegian Institute of Public Health, Division of Epidemiology, Department of Mental Health, Oslo, Norway

## Abstract

**Background:**

Most western countries have disability benefit schemes ostensibly based upon requiring (1) a work inhibiting functional limitation that (2) can be attributed to a diagnosable condition, injury or disease. The present paper examines to what extent current practice matches the core premises of this model by examining how much poorer the perceived health of disability benefit recipients is, compared to the employed and the unemployed, and further to examine to what extent any poorer perceived health among benefit recipients can be attributed to mental or somatic illness and symptoms.

**Methods:**

Information on disability benefit recipiency was obtained from Norwegian registry data, and merged with health information from the Hordaland Health Study (HUSK) in Western Norway, 1997–99. Participants (N = 14 946) aged 40–47 were assessed for perceived physical and mental health (Short Form-12), somatic symptoms, mental health, and self reported somatic conditions and diseases treated with medication. Differences associated with employment status were tested in chi-square and t-tests, as well as multivariate and univariate regression models to adjust for potential confounders.

**Results:**

Recipients of disability benefits (n = 1 351) had poorer perceived physical and mental health than employees (n = 13 156); group differences were 1.86 and 0.74 pooled standard deviations respectively. Self reported somatic diagnoses, mental health and symptoms accounted for very little of this difference in perceived health. The unemployed (n = 439) were comparable to the employed rather than the recipients of disability benefits.

**Conclusion:**

Recipients of disability benefits have poor perceived health compared to both the employed and the unemployed. Surprisingly little of this difference can be ascribed to respondents' descriptions of their illnesses and symptoms. Even allowing for potential underascertainment of condition severity, this finding supports the increasing focus on non-disease oriented contributing factors. Rehabilitation efforts aiming at return to work should have a strong focus on the patients' perceptions of their health in addition to symptom relief and social factors.

## Background

Despite public health improvements on several parameters over recent decades, many western countries report an increasing number of people living on health related benefit schemes, leading to calls for welfare systems reform [1-3]. A recent review concluded that research on most aspects of disability benefits and sickness absence is limited, with little scientific evidence to inform reforms. This lack of knowledge also affects legislation and clinical decisions within the present schemes [4,5].

National policies for awarding disability benefits vary, but ostensibly all adhere to a medical model requiring a) work inhibiting functional limitation that b) can be attributed to an acknowledged condition, injury or disease [1]. The degree of limitation was formerly based on objective measures in interaction with actual work characteristics: For a craftsman, a rate derived from what limb was lost and how essential this was for his work[6]. At present, most disability expenditure is for musculo-skeletal and psychiatric disorders, both generally relying on subjective reports of functional limitations and symptoms[7]. Rising levels of claimants, despite generally better population health indices, have led to attempts to elucidate social risk factors such as area level effects and education that interplay with medical factors[8].

Evaluations of functional limitations from health complaints or disease are largely based on patients' perceived health. This subjectivity has nurtured alternative explanations to the medical model: Pull-factor theories hypothesize that benefit schemes might have become too generous and attract recipients on insufficient grounds[6,9], while push-factor theories focus on factors that expel people from work, through either health-problems incompatible with available jobs, or structural economic changes undermining financial job-security and forcing the individual into a sick-role or unemployment as alternative sources of income [10-12]. To the extent that health is involved, there is no conflict between the push-model and the existing practice for award of disability pension. The attraction model (hypothesizing the role of "pull-factors" and discounting health) challenges both the push-model and the traditional medical perspective.

Among the few empirical investigations in this area, a recent report from the Organisation for Economic Cooperation and Development (OECD) conclude that, on average, 1/3 of disability benefit recipients do not classify themselves as disabled, suggesting inclusion errors in disability schemes[1]. In a recent Swedish study it was reported that, among those receiving disability pensions for musculoskeletal disorders, 27.7 % rated their health as "good" or "fairly good" three to eight years later[13]. In these studies, self ratings of disability among disability pension recipients were not contrasted with ratings among the general population or other marginalized groups, e.g. the unemployed, who according to a recent meta-study also experience reduced physical and psychological well-being[14].

The aim of the present study was to investigate the two core premises of the current model through a) comparing perceived health of disability benefit recipients with both employees and unemployed, and b) examine to what degree self-reported medical conditions and symptoms explain the difference. We hypothesised that these medical conditions and symptoms would account for little of any difference found in perceived health.

## Methods

### Population and data material

The Hordaland Health Study 1997–1999 (HUSK) was a joint epidemiological research project carried out by the National (Norwegian) Health Screening Service in collaboration with the University of Bergen. The base population included 29 400 individuals in Hordaland County in western Norway born 1953–57, aged 40–47 at the time of the data collection. Data were collected by questionnaires and clinical examinations. A total of 18 581 (8 598 men and 9983 women) both answered the first questionnaire and came to the clinical examinations, yielding a participation rate of 63 % (57 % for men and 70 % for women).

After the clinical examinations, the second questionnaire was distributed and prompted for return by mail. Due to non-response to one or more of the variables in the second set of questions, 2893 individuals were excluded, and 742 individuals, who carried no information on either employment or benefits in the registry, were classified as "inactive" and also excluded from the study. Thus, the final population consisted of 14 946 individuals (table 1).

### Employment status and receipt of benefits

In the health survey there were items on full time work, full time domestic work, education or military service, being unemployed or laid-off. Those who responded positively to either of the first three categories, confirmed shift-work or reported more than any paid work during the week, constitute the group of "*employed*" (n = 13 156). The 1 790 benefit recipients comprise two groups: recipients of 1) *unemployment *benefits and 2) *disability benefits*; the latter group comprising long-term sickness absentees (>14 days of general practitioner (GP) warranted sickness absence, n = 601), recipients of medical or occupational rehabilitation benefits (n = 250), and those receiving permanent disability pensions (n = 500). These three were collapsed into one group as differences between them were minor and considered trivial in this context (data not shown). Information on benefits was confirmed from the National Insurance Administration (NIA) and was merged to the health survey by Statistics Norway through the national identification number. Duration and type of benefit is registered and allow for accurate calculations of benefit spells. Cases with more than one suitable category (e.g. both working and receiving a possible less than 100 % compensation disability pension), were appointed to the alleged most severe and permanent benefit category.

### Perceived health

The outcome variables 'perceived physical and mental health status', were measured using the self report Short Form-12[15,16]. This shorter version of the SF-36 is recommended for large population surveys such as the HUSK[16]. Weighted summation provides summary scores for *perceived mental health *and *perceived physical health*. Out of the total twelve items, eight enquire directly on functional limitations due to health. The measurement has been standardized according to US norm data[16], with a mean score of 50 (SD 10). All results are presented as un-standardized regression coefficients for group differences. Missing values on single items were estimated from the valid responses and the linear regression coefficients predicting the score on the particular missing item(s) derived from those who had completed all items. This procedure was relevant for 1 069 participants, of which 831 had only one item substituted.

### Health

#### Somatic conditions

Questions on somatic diagnoses were framed in the form of: "Do you have or have you had (one of the following)", coronary infarction, stroke, diabetes, asthma, multiple sclerosis, chronic bronchitis, osteoporosis, or fibromyalgia. A positive response on one or more of these items was considered *self reported diagnosis *positive. In addition, participants were asked if they used any medication the previous day, and if so, for which condition. From these responses, a team of physicians appointed appropriate ICPC-diagnoses according to *ATC-classifications*, producing a continuous variable indicating number of conditions for which the person is taking medication.

#### Mental health

Anxiety and depression symptoms were assessed with the Hospital Anxiety and Depression Scale (HADS), which contain seven items each on cognitive symptoms of anxiety disorder and depression[17]. In recent literature review, HADS showed good case-finding properties for anxiety and depression in primary care patient populations and hospital settings[18] and is more accurate than GPs[19]. The HADS-scores are used as continuous variables, reflecting increasing *anxiety *and *depression *symptom load.

#### Somatic symptoms

Participants were also asked about frequency of 17 common symptoms from different organ systems in accordance with the ICD-10 Research Criteria for F45 Somatoform Disorders[20], on a five point likert scale labelled; "almost never, rarely, sometimes, often and almost always", rated 0–4. The items were summed and comprise the variable *organ system symptoms *that is used as a continuous variable, with increasing levels reflecting higher symptom load. Total symptom scores are often used in both research and clinical practice to determine severity levels, especially for mental health conditions. In addition, participants were asked if they had been troubled with muscle pain and/or stiffness in muscles or joints continuously for over three months during the last year. If positive, they were further asked to reply to which of ten suggested joints or body areas was affected. This was included as the continuous variable *muscle pain*, ranging from nil to ten where increasing numbers indicate increasing severity. Finally, information on *sleep problems *last 30 days was self-reported on a four point likert scale. For all three variables, a higher score reflect more health problems.

### Socio-demographic and behavioural factors

Self reported annual household *income *was measured by one item and coded in three categories from no income to more than NOK 500 000 (approximately € 60 000). Level of *education *was reported in four categories from less than seven years of schooling up to at least 4 years of higher education in college/university. *Marital status *was self-reported and dichotomized as being single or not. Self reported weekly consumption of *alcohol *units was entered as a continuous variable, as was body mass index (*BMI*), calculated from body weight by squared height from the clinical examinations.

### Statistical analysis and models

Associations between work and benefit status and perceived health was examined through comparing means of SF-12 scores in linear regression models run in SPSS 13.0, with the three variables indicating each employment-status entered as independent variables. Age adjustment was not considered due to restriction in variance.

In gender adjusted multivariate analyses, blocks of theoretically related variables were entered sequentially in an *a priori *determined order, and finally in a fully adjusted model. Somatic conditions were entered first, as it was presumed to be an important cause of disability benefits and to avoid overestimating subsequent effects of mental health and somatic symptoms. The mental health variables were entered as the second block, and then the somatic symptoms variables which might be products of either somatic conditions or mental health problems. The same hierarchical model was employed for both perceived mental and physical health. Socio-demographic and behavioural factors were entered last, so that any effect of these adjustments should indicate social inequality beyond health. To examine potential confounding effects from specific health and socio-demographic variables, each of these was entered in separate univariate analyses after gender adjustment. Results are reported with a 95 % confidence interval for the estimates, significance level p < .05. Testing for demographic differences was done by Pearson Chi square and for crude perceived health by t-tests.

### Ethics

The study protocol was approved by the Regional Committee for Medical Research Ethics, Western Norway and by the Norwegian Data Inspectorate.

## Results

Of the 14 946 participants, 88.0 % (87.5–88.5) were employed, 2.9 % (2.7–3.2) unemployed and 9.1 % (8.6–9.6) were disability benefit recipients. The overall sample means on the SF-12 scales were 50.10 (SD 8.18) for perceived physical health and 52.54 (SD 8.56) for perceived mental health, similar to US norms. Among those 10835 who were invited to the health study, but did not attend, a greater proportion, 18.5 % (18.1–18.9), were recipients of unemployment or disability benefits at the time. Of the 3 619 who attended, but failed to complete the necessary items in the study, 13.3 % (12.1–14.4) received unemployment or disability benefits.

In pair-wise comparisons of the groups' demographic profiles, the unemployed and the disability benefit recipients were different from the employed on all parameters (p < .001). However, there were no significant differences between the unemployed and the disability benefit recipients in terms of gender (p = .66), income (p = .52) and marital status (p = .24), whilst they were different on level of education (p < .05). In contrast to these demographic similarities, the unemployed were more similar to the employed in terms of perceived health, which was better than among disability pensioners (all group comparisons p < .001) (Table 1).

**Figure 1:**
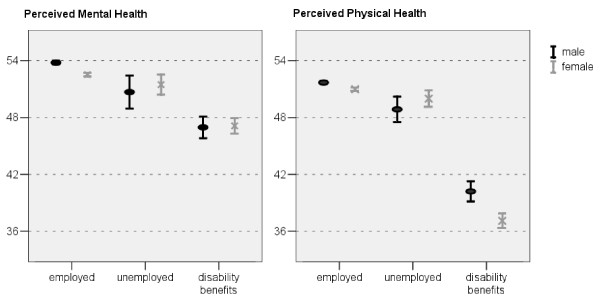
Perceived mental and physical health across employed, unemployed and disability benefit recipients.

Disability benefit recipients perceived their health to be much poorer than employees (Figure 1). This difference was more pronounced in perceived physical health, where they scored 1.86 pooled standard deviations lower than the employed. On the perceived mental health their average was 0.74 pooled standard deviations lower. The perceived health of the unemployed was closer to the employed than to those on disability benefits with scores of 0.26 and 0.25 pooled standard deviations below employees on perceived physical health and perceived mental health respectively.

In the gender adjusted blockwise linear regression, adjustment for "somatic conditions" had no effect on the differences in perceived mental health between the employed and disability benefit recipients, while "mental health" explained more than half of the difference, and left the association statistically non significant. Adding "somatic symptoms" had more explanatory power, but adjusting for demographic variables added nothing further.

The gender adjusted differences in the perceived physical health were surprisingly not attenuated by neither "somatic conditions" nor "mental health": the two blocks together only explained 8.5 % of the group difference. However, "somatic symptoms" was an important confounder and attenuated the perceived physical health difference by a further 27.7 %. The fully adjusted model showed a small confounding effect of "socio-demographic and behavioural factors" on top of the health variables. The differences between employed and the unemployed were non-significant in the fully adjusted models (Table 2).

In the univariate analysis only *organ system symptoms *and in particular *muscle pain *demonstrated a reasonable confounding effect on the association of benefit status and perceived physical health. The association of perceived mental health and employment status was also attenuated by these variables, as well as by *depression *and *anxiety*, *sleep problems *and *income *(Table 3).

## Discussion

### Main results

The group differences in perceived health (both mental and physical) between the employed and disability benefit recipients were substantial, whilst the perceived health of unemployed was comparable to that of the employed rather than those on disability benefits. The markedly more pronounced difference in perceived *physical *than *mental *health was only modestly attributable to somatic conditions, mental health or somatic symptoms. Socio-demographics and health behaviours had little additional confounding influence upon these strong associations.

### Strengths and limitations

The present study has several strengths. The classifications of benefits were obtained from highly reliable national registries. The design of the study, employing several sources of data in a health context, reduces biases from selective symptom report to gain or avoid access to benefits as the participants were unaware of the outcome. The Short Form-12 is developed for use in general populations, and the semantics of the items resemble likely questions in clinical settings to determine patients' health perceptions relating to work ability. Furthermore, it differentiates between mental and physical aspects of perceived health. The study covers somatic conditions, mental health and somatic symptoms that encompass the most prevalent diseases and illnesses in benefit recipiency, as well as socio-economic variables relevant for both health and benefit recipiency[8]. Finally, the response-rate of the study was satisfactory, the included age span is highly relevant as participants potentially have a number of years left as members of the work-force, and the population was drawn from the general population in a representative area with both urban and rural communities.

The study also has some limitations. The non-response rate among the benefit recipients was higher than for the employed. This could cause an underestimation of the true differences between the groups, although usually non-responders are more functionally limited. The list of symptoms and conditions is not complete and relies upon self report, potentially limiting our ability to adjust fully for a confounding effect of health. Residual confounding from random measurement errors is probably the most important limitation, resulting in underestimation of the proportion of group difference in perceived health attributable to somatic and mental health conditions and symptoms. Type of employment can have an effect on health[21], and by attributing any confusing exposure to the benefit group we have used a conservative approach, likely to reduce any observed differences in perceived health. Any confounding effect of income may be overestimated due to circularity between income and employment status. Finally the minimisation of the age range prevents analysing interactions with age, or generalising to other age groups.

### Interpretation

Disability benefits are administered according to *policies *which require a diagnosable medical condition resulting in work-related *impairment *for granting disability benefits. This study offers empirical data examining to what extent there are differences in functional limitations (as measured by SF-12 *perceived health*) between the employed and disability benefit recipients, and to what extent these differences can be attributed to somatic conditions and mental and somatic symptoms regardless of aetiology

The first criterion, that there must be a functional limitation, was supported from the poorer perceived health among disability benefit recipients. This very strong association, particularly with physical health, suggests the current system is successful in identifying those less capable of working. On the other hand, reverse causality may explain these findings if deprivation of normal role functioning is in itself disabling and that perceived health decreases following disability benefit award[7,22]. Our results suggest that such a process may operate as a much smaller, but significant, reduction in perceived health is observed among the unemployed who, in a similar vein, are deprived of normal role functioning. One explanation is that cognitive processes among benefit recipients decrease perceived health to match their present status as exempted from work due to "deteriorated health"[23]. Causal explanation aside, there is support from other studies that the severity and longevity of sickness absence is associated with adverse outcomes and can predict grave endpoints like mortality[24].

The second criterion, that this reduced ability to work must be ascribed to an acknowledged diagnosis was only partially supported. Anxiety and depression scores (a proxy for diagnoses[19]) alone explained more than half of the difference in perceived mental health between the disability benefit recipients and the employed. However, this criterion was not supported for perceived physical health, as somatic diagnoses barely attenuated the group difference. It might be argued that these adjustments do not account for the severity of a condition whereby the severity of conditions in the disabled is greater than that in those still able to hold down a job. Severity might be approximated by the total symptom count, certainly in the case of mental symptoms where counts are frequently used as measures of condition severity, and to some extent also in physical conditions. Somatic symptoms did have attenuating accounting for approximately a quarter of the variance. The substantial residual differences are unlikely to be completely explained by misclassification in the responses of the employed and disability pension recipients, or enormous other health related differences not detected in the health survey.

The cross sectional nature of the study cannot exclude that some of the unexplained difference could be a derivative of elevated symptoms levels at the time of applying for benefits that later have regressed towards a normal level either through a natural course, treatment or that the induced absence from work has ameliorated the symptoms. If the latter is the case, careful evaluation of whether re-entry to work is likely to cause the symptoms to remit is needed. If as suggested, cognitive changes in perceived health is caused by changes in work role, and these changes persists beyond symptom relief, negative health perceptions that do not self-resolve needs to be specifically addressed. From studies on working age populations, it is reported that measures of mental and physical health are the most important determinants of self-rated health[25]. The results of the present study suggest that additional factors are important in explaining the worse perceived health among disability benefit recipients.

To our knowledge, the importance of perceived health in disability benefits has rarely been subject to empirical examination. Supporting evidence is found in a study where a single dichotomous item of self-rated health strongly predicted disability pension over an eleven year follow-up among men. As in the present study, adjusting for baseline somatic disorders, musculoskeletal disease, mental disorders and medication use, did little to attenuate this risk[26].

If even some of the large difference found in perceived *physical *health between individuals claiming disability benefits and the employed cannot be attributed to somatic conditions or mental and somatic symptoms, this implies that interventions aimed solely at medical problems amongst benefit recipients would have a limited effect on return to work for many. Thus, in (medical) rehabilitation and treatment efforts to alleviate work disability, patients' own perceptions of health and ability to work should be addressed in addition to symptom relief. In the UK work rehabilitation trials it was found that the health status of the individual had little predictive power for identifying those likely to return to work whilst individual's perception of their likelihood of returning and job satisfaction were strong predictors[27]. Following on from observations that health perceptions were important predictors of return to work after a myocardial infarction, a small RCT demonstrated that a short intervention designed to alter individuals health perceptions improved the likelihood of post-MI return to work[28].

These results are relevant for the current dissonance in benefit practice, where physicians formally are appointed as gate keepers, but rarely exert their authority in confronting patients motivated for disability benefits [29-31], generally taking what patients say at face value The impact of sick-roles[32] and personal attributions about work ability and prospective return to work needs further attention, as do factors beyond health influencing disability benefit influx, including both push- and pull-factors[33].

## Conclusion

The perceived health among disability benefit recipients is markedly poorer compared to both employed and the unemployed. This difference is largest in perceived *physical health*, but less than 1/3 of this difference is explained by the self reported diagnoses and symptoms. Symptoms of anxiety and depression account for 2/3 of the difference between the employed and the unemployed in their perceived mental health, and more than 1/2 of the difference between the employed and the disability benefit recipients. Interestingly, the unemployed resemble the disability benefit recipients in terms of demographic characteristics albeit their perceived health is more like that of the employed. The implications of these findings for policy are several; according to current policy disability benefits are awarded for impaired work ability due to a diagnosable condition. Disability benefit recipients perceive their health to be poor, but surprisingly little of this can be attributed to the symptoms and diagnosable conditions reported. This indicates that an individual's perception of ones health takes many more factors into account than could be identified in this survey. This may reflect health behaviours e.g. smoking and fitness, varying social acceptability of health complaints and subgroup or geographical variation in health assessment. Finally the effect of being given a label of permanent disability may be more deleterious to an individual's self appraisal than previously thought. This suggests that rehabilitation efforts aiming at return to work should have a strong focus on the patient's perceived health in addition to symptoms relief.

## Competing interests

The author(s) declare that they have no competing interests.

## Authors' contributions

SO conceived of the study, performed data analysis, drafted the manuscript and coordinated the study. NG participated in conceiving the study, interpretation of the results and drafting the manuscript. JGM participated in conceiving the study, acquisition of data and drafting the manuscript. LEA revised the manuscript for important content. AM participated in designing the study, data analysis and drafting the manuscript. All authors read and approved the final manuscript.

## Pre-publication history

The pre-publication history for this paper can be accessed here:



## Figures and Tables

**Table 1:** Demographic variables and means of perceived health across employed, unemployed and disability benefit recipients

Variable	Employed	Unemployed	Disability Benefits
N	13156	439	1 351
% of sample	88.0	2.9	9.1

Gender (n/%)			
Female	6754/51.3	293/66.7	886/65.6
			
Education (n/%)‡			
Primary	2051/15.6	126/28.7	441/32.6
Secondary	6041/45.9	228/51.9	607/44.9
1–3 yr higher	2662/20.2	41/9.3	184/13.6
≥ 4 yr higher	2402/18.3	44/10.0	119/8.8
			
Income (n/%)‡			
≤ 249 999	3681/28.0	210/47.8	642/47.5
250000 – 499 999	6001/45.6	196/44.6	585/43.3
500000 ≤	3474/26.4	33/7.5	124/9.1
			
Marital Status (n/%)			
Single	3129/23.8	159/36.2	532/39.4

Perceived Physical Health (mean)	51.34	49.62	38.17
			
Perceived Mental Health (mean)	53.15	51.23	47.07

‡ Some of the percentages do not add up to 100 due to rounding

**Table 2:** SF-12 Perceived Mental and Physical Health by employment status adjusted for hierarchical blocks of confounders

		Perceived Mental Health	
Blockwise adjustments	Employed	Unemployed	Disability Benefits
A. Gender	0.0 (ref)	-1.8 (-2.6, -1.0)	-5.9 (-6.4, -5.5)
B. A + Somatic Conditions	0.0 (ref)	-1.8 (-2.6, -1.0)	-5.8 (-6.4, -5.4)
C. B + Mental health	0.0 (ref)	-0.6^A ^(-1.2, 0.1)	-2.6 (-3.0, -2.2)
D. C + Somatic symptoms	0.0 (ref)	-0.5^A ^(-1.1, 0.1)	-2.3 (-2.7, -1.9)
E. D + Socio-demographics/Fully adjusted model	0.0 (ref)	-0.5^A ^(-1.1, 0.1)	-2.3 (-2.7, -1.9)

		Perceived Physical Health	
Blockwise adjustments	Employed	Unemployed	Disability Benefits

A. Gender	0.0 (ref)	-1.6 (-2.3, -0.9)	-13.0 (-13.5, -12.6)
B. A + Somatic Conditions	0.0 (ref)	-1.5 (-2.2, -0.9)	-12.5 (-12.9, -12.0)
C. B + Mental health	0.0 (ref)	-1.3 (-2.0, -0.6)	-11.9 (-12.3, -11.5)
D. C + Somatic symptoms	0.0 (ref)	-0.8 (-1.4, -0.3)	-8.6 (-9.0, -8.2)
E. D + Socio-demographics/Fully adjusted model	0.0 (ref)	-0.7^A ^(-1.2, 0.1)	-8.5 (-8.8, -8.1)

The employed as reference in multivariate regression, unstandardized regression coefficients and 95 % confidence intervals, ^A ^= Not statistically significant; p > .05

**Table 3:** SF-12 Perceived Mental and Physical Health by employment/benefit status with separate adjustments for confounders

		Perceived Mental Health		Perceived Physical Health	
Variable	Employed	Unemployed	Disability Benefits	Unemployed	Disability Benefits
Gender	0.0 (ref)	-1.8 (-2.6, -1.0)	-5.9 (-6.4, -5.5)	-1.6 (-2.3, -0.9)	-13.0 (-13.5, -12.6)

Self reported diagnoses	0.0 (ref)	-1.8 (-2.6, -1.0)	-5.9 (-6.4, -5.4)	-1.5 (-2.2, -0.9)	-12.8 (-13.2, -12.4)
ATC- Classification	0.0 (ref)	-1.8 (-2.6, -1.0)	-5.9 (-6.3, -5.4)	-1.6 (-2.3, -0.9)	-12.8 (-13.1, -12.3)
Anxiety	0.0 (ref)	-0.9 (-1.6, -0.3)	-3.3 (-3.7, -2.9)	-1.5 (-2.2, -0.8)	-12.7 (-13.1, -12.3)
Depression	0.0 (ref)	-0.7 (-1.3, -0.0)	-3.3 (-3.7, -2.9)	-1.4 (-2.0, -0.7)	-12.5 (-12.9, -12.1)
Organ system symptoms	0.0 (ref)	-1.2 (-2.0, -0.5)	-3.8 (-4.3, -3.3)	-1.0 (-1.6, -0.4)	-10.9 (-11.3, -10.5)
Muscle pain	0.0 (ref)	-1.6 (-2.4, -0.8)	-5.0 (-5.4, -4.5)	-1.0 (-1.6, -0.4)	-9.3 (-9.7, -9.0)
Sleep problems	0.0 (ref)	-1.3 (-2.0, -0.5)	-4.0 (-4.4, -3.5)	-1.4 (-2.0, -0.7)	-12.2 (-12.6, -11.8)
Income	0.0 (ref)	-1.3 (-2.1, -0.5)	-5.5 (-6.0, -5.0)	-1.3 (-2.0, -0.6)	-12.8 (-13.2, -12.4)
Education	0.0 (ref)	-1.9 (-2.7, -1.1)	-6.0 (-6.5, -5.6)	-1.3 (-1.9, -0.6)	-12.7 (-13.1, -12.3)
Marital Status	0.0 (ref)	-1.5 (-2.3, -0.7)	-5.6 (-6.1, -5.1)	-1.6 (-2.3, -0.9)	-13.1 (-13.5, -12.7)
Alcohol	0.0 (ref)	-1.7 (-2.5, -1.0)	-6.0 (-6.4, -5.5)	-1.6 (-2.3, -0.9)	-13.0 (-13.4, -12.6)
BMI	0.0 (ref)	-1.8 (-2.6, -1.0)	-6.0 (-6.5, -5.5)	-1.4 (-2.1, -0.8)	-12.8 (-13.3, -12.4)

The employed as reference in linear regression analysis, unstandardized regression coefficients and 95 % confidence intervals
